# Rapid identification of anti-idiotypic mAbs with high affinity and diverse epitopes by rabbit single B-cell sorting-culture and cloning technology

**DOI:** 10.1371/journal.pone.0244158

**Published:** 2020-12-21

**Authors:** WeiYu Lin, Wei-Ching Liang, Trung Nguy, Mauricio Maia, Tulika Tyagi, Cecilia Chiu, Kam Hon Hoi, Yongmei Chen, Yan Wu

**Affiliations:** 1 Department of Antibody Engineering, Genentech Inc., South San Francisco, CA, United States of America; 2 DevSci BioAnalytical Sciences, Genentech Inc., South San Francisco, CA, United States of America; King's College London, UNITED KINGDOM

## Abstract

The proactive generation of anti-idiotypic antibodies (anti-IDs) against therapeutic antibodies with desirable properties is an important step in pre-clinical and clinical assay development supporting their bioanalytical programs. Here, we describe a robust platform to generate anti-IDs using rabbit single B cell sorting-culture and cloning technology by immunizing rabbits with therapeutic drug Fab fragment and sorting complementarity determining regions (CDRs) specific B cells using designed framework control as a negative gate to exclude non-CDRs-specific B cells. The supernatants of cultured B cells were subsequently screened for binding to drug-molecule by enzyme-linked immunosorbent assay and the positive hits of B cell lysates were selected for cloning of their immunoglobulin G (IgG) variable regions. The recombinant monoclonal anti-IDs generated with this method have high affinity and specificity with broad epitope coverage and different types. The recombinant anti-IDs were available for assay development to support pharmacokinetic (PK) and immunogenicity studies within 12 weeks from the start of rabbit immunization. Using this novel rapid and efficient in-house approach we have generated a large panel of anti-IDs against a series of 11 therapeutic antibody drugs and successfully applied them to the clinical assay development.

## Introduction

Therapeutic antibodies are a rapidly-growing class of drugs with strong affinity, high specificity, prolonged half-life, and strong pharmacological potency. These properties have made therapeutic antibodies a well-established platform for the development of efficacious treatments against a wide-variety of human diseases [1]. Their application areas are not only limited to standard immunoglobulin G (IgG) format but can also be extended to pursue “undruggable” targets under novel formats including antibody fragments (scFv, VHH), drug conjugates, and bispecifics [2–5]. With numerous antibody therapeutics (Ab1) being continuously advanced into preclinical development and clinical trials, the demand for high-quality critical reagents for bioanalytical support of those programs is high. In that context, anti-idiotypic reagent antibodies (anti-IDs or Ab2) have become one of the preferable options for bioanalytical applications at pre-clinical and clinical stages. Common approaches used to generate these types of reagent antibodies against Ab1 include *in vivo* hybridoma technology, *in vitro* phage-displayed immunized or synthetic antibody libraries [6–8]. In our experience these technologies work reasonably well for generation of anti-IDs but require extended timelines and often yield low-number panels of super-high affinity monoclonal antibodies (mAbs) that lack epitope-binding diversity.

Rabbit mAbs are known for their high affinity and diversity making them a potentially useful source of highly specialized research reagents in bioanalysis programs for therapeutic candidates [9]. In the past rabbit mAbs were generated using hybridoma technology [10]; currently however, discovery is driven by a more robust single B cell culture and cloning method [11–13].

High quality anti-IDs particularly Ab2 that are CDR-specific are critical to develop sensitive, specific and robust PK and anti-drug antibody (ADA) assays to support pre-clinical and clinical studies in drug development programs. Here, we have applied an optimized rabbit single B cell sorting-culture and cloning method for Ab1 CDRs-specific anti-IDs discovery which include four phases: 1) IgG^+^ B cells enrichment before sorting, 2) guided selection using a designed Ab1 framework control Fab (Ab1^ctrl^ Fab) as a “negative gate” to exclude Ab1 framework-specific B cells, 3) integrated robotic system to enhance screening throughput of B cells cultured supernatants, and 4) preserved B cells in screening for follow-up rapid cloning and recombinant IgG expression. Using this novel approach, we have successfully completed 11 projects (A-K) and generated high sequence diversities (>55%) with a broad affinity range (single digit pM to hundredth nM) of anti-IDs against unique Ab1, regardless of the number of clones being identified in the process (**Table 1**). In this paper we will highlight project E as an illustration of our effort spanning from early discovery phase to final anti-IDs characterization.

**Table 1:** Framework control, molecular cloning and binding affinity summary in multiple anti-IDs project campaigns.

Project	Ab1 light chain			Ab1 heavy chain			Ab1^Ctrl^	Anti-IDs characterization			
	Human framework germline	Closest human framework	Identity (%)	Human framework germline	Closest human framework	Identity (%)	Human framework control	Ab1 CDRs specific clones	Molecular cloning		Affinity range (K_D_: nM)
									Cloned	Uniqueness (%)	
A	hIGKV1-39	K1	98	hIGHV1-46	H1	98	K1H1	146	96	78	0.002–71
B	hIGKV4-01	K4	99	hIGHV1-46	H1	99	K4H1	75	75	59	0.04–8
C	hIGKV1-16	K1	99	hIGHV4-59	H4	93	K1H4	901	48	88	0.01–44
D	hIGKV4-01	K4	99	hIGHV1-03	H1	99	K4H1	48	48	55	0.003–4
E	hIGKV1-16	K1	100	hIGHV3-23	H3	98	K1H3	34	34	71	0.004–6
F	hIGKV1-05	K1	93	hIGHV1-02	H1	89	K1H1	48	48	83	0.03–202
G	hIGKV1-16	K1	96	hIGHV3-48	H3	99	K1H3	37	37	76	0.15–4
H	hIGKV1-12	K1	93	hIGHV1-46	H1	98	K1H1	796	48	92	0.016–1
I	hIGKV1-39	K1	98	hIGHV1-46	H1	99	K1H1	79	42	69	0.007–167
J	hIGKV1-12	K1	100	hIGHV3-23	H3	96	K1H3	659	48	81	0.001–2
K	hIGKV2-28	K2	98	hIGHV3-23	H3	96	K2H3	967	96	75	0.006–8

The framework germlines in 11 unique Ab1 projects and their corresponding closest human framework control chosen to guide selection are listed. The identity (%) of light and heavy chain for each project were determined from sequence alignment of Ab1 framework germline with the closest human framework. Part or all of the Ab1 CDRs-specific anti-IDs were molecular-cloned to determine their sequence uniqueness shown in %. The unique clone here was defined by >10% amino acid difference in CDRs sequences. The binding affinities for all unique anti-IDs against Ab1 Fab at each project were measured by Biacore SPR and listed as a range from single digit pM to hundredth nM. Ab1, antibody therapeutics.

With the optimized single B cell sorting-culture and cloning technology described herein, we have expanded capability of generating large panels of anti-IDs routinely and with a high degree of success. Compared to previous antibody discovery approaches, this new platform consistently and effectively delivers high affinity anti-IDs with high-degree of Ab1-CDRs specificity and more diverse epitopes in a fraction of time providing tangible benefits to bioanalytical programs supporting recombinant therapeutic antibody projects.

## Materials and methods

### Rabbit immunization and peripheral blood IgG B cell enrichment

Three New Zealand White (NZW) rabbits purchased from Western Oregon Rabbit Company (WORC) were immunized with Ab1 Fab by a local contract research organization. All animal procedures were performed in accordance with the Animal Welfare Act. Ethical review of all animal activity was approved and performed by the contract research organization's Institutional Animal Care and Use Committee (Josman LLC IACUC), and all animal procedures were performed in accordance with Animal Care and Use Protocol (ACUP) that was approved by the IACUC (JLP-003.019). Appropriate anesthetics (isoflurance) and analgesics were used in study procedures when needed, as approved in the IACUC protocol. All animals were humanely euthanized using appropriate euthanasia agents (Euthasol/Beuthanasia) and methods approved in the IACUC protocol. After receipt of animals, acclimation for at least 48 hours prior to initiation of study. Rabbits were individually housed with food and water, any materials in contact with the rabbits were of lab animal industry standards. All procedures listed in the protocol involving live animals (including euthanasia and anesthesia) were performed by trained, qualified personnel. For immunization or blood collection procedures, rabbits were appropriately anesthetized as required in the animal care and use protocol. The injection sites will be shaved and aseptically prepped with isopropyl alcohol skin prep. Each rabbit was immunized with 400 ug of Ab1 Fab formulated with Complete Freund's adjuvant (CFA) in a 1:1 mix by subcutaneous (SC) and intradermal (ID) injections at multiple sites. Initial immunization was followed by three boosts of 200 ug Ab1 Fab formulated with Incomplete Freund's adjuvant (IFA) in 1:1 mix via SC injection as approved in the protocol. After procedures rabbit was recovered from anesthesia and monitored continuously for signs of lethargy until fully ambulatory and moving around normally. Up to 20 ml of blood was collected from the central ear artery with a butterfly needle. The rabbits were monitored closely for signs of infection, ulceration or granulomas at the injection sites. Followed by the 3rd boost, the antibody titers were evaluated. Rabbits developed anti-Ab1 Fab polyclonal sera titer during the immunization period were measured by standard ELISA protocol. A 96-well Nunc microtiter plate was coated with Ab1 Fab (1ug/mL) in the coating buffer (0.05M sodium carbonate, pH9.6) overnight at 4°C. The plate was then blocked with assay buffer (1x PBS, 0.5% BSA and 0.05% polysorbate 20) before incubating with serial dilution of rabbit serum for an hour. Bound antibodies were detected by goat anti-rabbit IgGs horseradish peroxidase conjugate (Sigma) with TMB substrate (Surmodics, Inc.); color development was and ceased with Stop Solution (Surmodics, Inc.) for 5 min, prior to optical density (OD) reading at 650nm. After antibodies of interest were identified, rabbits were euthanized using euthanasia solution approved in the IACUC protocol.

Rabbit PBMCs were isolated by density centrifugation of whole blood using Lympholyte®-M (CL5030, Cedarlane Labs). IgG B cell were negatively selected through MACS Column (130-042-401, Miltenyi Biotec). Unwanted cells were targeted to removal with antibody cocktails containing anti-rabbit CD11b antibody, anti-rabbit T-lymphocyte antibody (MCA802GA, MCA800GA, AbD Serotec, BioRad), and anti-rabbit IgM (550938, BD Bioscience). In the presence of magnetic beads only the cells that are bound to the antibodies could be attached to the beads when passing them through the column with applied magnetic field. The unbound IgG^+^ B cells were able to pass through the column and got enriched in the process.

### Ab1 and Ab1 framework control (Ab1^ctrl^) Fab preparation and fluorescence labeling

The Fab fragments (Ab1 Fab) of therapeutic Ab1 IgG antibodies were prepared by lysyl endopeptidase (129–02541, Wako Chemicals, Inc.) digestion, followed by protein *L*-agarose column purification [14]. The Ab1^ctrl^ Fabs were designed by grafting LC and HC CDRs of irrelevant anti-gD mAb (5B6; Genentech), respectively, onto four human LC (hIGKV1/V2/V3/V4 or K1/K2/K3/K4) and four human HC (hIGHV1/V2/V3/V4 or H1/H2/H3/H4) frameworks which were determined by selecting the most prevalent amino acid residue at a given position of human framework germline genes most frequently used in the natural human antibody repertoires [15, 16]. A total combination of 16 Ab1^ctrl^ Fab fragments (KnHn, n = 1/2/3/4) were transiently expressed in Expi293F cells (A14528, Thermal Fisher Scientific) followed by Protein G affinity chromatography (17088601, GE Healthcare) purification, as reported previously [17]. For fluorescence labeling, the Ab1 and the corresponding Ab1^ctrl^ Fab fragments were conjugated with *R*-phycoerythrin (703–0003, RPE; Innova Biosciences) and allophycocyanin (705–0030, APC; Innova Biosciences), respectively, according to the manufacturer’s instructions.

### Ab1 CDRs-specific IgG^+^ single B cell sorting, culture, and screening

The enriched rabbit IgG B cells were stained with mixed FITC-labeled goat anti-rabbit IgG antibody (STAR121F, Serotec, BioRad), RPE-labeled Ab1 Fab, and APC-labeled Ab1^ctrl^ Fab in the staining buffer (Phosphate-buffered saline with 2% fetal bovine serum). A Becton Dickinson FACS Aria sorter was used for Ab1-specific IgG^+^ B cell sorting. The FACS gating strategy used for identify Ab1-specific IgG B cells is as following: after gating lymphocytes (SSC-A vs FSC-A) and singlet (FSC-H vs FSC-A), live cells were gated by the negative of PI (Propidium Iodide). FITC labeled IgG^+^ B cells were identified and their Ab1-specific population (RPE-Ab1 Fab^+^ vs APC-Ab1^ctrl^ Fab^-^) was selected as sorting gate. The single IgG^+^ B cell from RPE^+^ and APC^-^ population was literally sorted into 96-well plate with supplemented RPMI 1640 culture medium and EL4-B5 feeder cells for 7 days cultivation at 37°C, as previously described [11].

The B-cell culture supernatants were then transferred via HTP robotic system (BioCel, Agilent) to screen Ab1 Fab and Ab1^ctrl^ Fab binding in a 384-well microplate by standard ELISA protocol, as described above. The Ab1 Fab^+^ and Ab1^ctrl^ Fab^-^ clones which were considered as Ab1 CDRs-specific Ab2 were then cherry picked from the original RLT lysis buffer (79216, Qiagen) treated source plates for molecular cloning.

### Ab2 rabbit antibody molecular cloning, sequence analysis, and expression

Total RNAs of Ab2 clones were first isolated using NucleoSpin 96 RNA Kit (740466.4, Machery & Nagel) according to the manufacturer’s instructions. The cDNA was prepared by reverse transcription of the mRNA from total RNA using SuperScript III First-Strand Synthesis SuperMix (18080400, Invitrogen). The V regions of individual rabbit B-cells were amplified through PCR reaction using AccuPrime Pfx SuperMix (12344040, Invitrogen), with forward and reverse primers designed to target variable light (VL) and heavy (VH) regions and then cleaned up using NucleoSpin 96 Extract II kit (740658.1, Machery & Nagel) as previously described [11]. The PCR fragments were cloned into the expression vectors encoding the respective rabbit IgG LC and HC constant regions by In-Fusion HD EcoDry Cloning Kit (638915, Takara) for sequence analysis and transfection to express recombinant rabbit IgGs [17]. Particularly for the sequence diversity analysis in project E, the CDR regions of both the VL and VH of the 24 unique clones were individually extracted and subsequently concatenated into a sequential residue string for each unique clone. The 24 residue strings were then aligned using Clustal W with both gap opening and extension penalties set at zero equivalent to local alignment [18]. In order to visualize the sequence similarity of the 24 clones, the aforementioned sequence alignment result was referenced to use with Neighbor Joining method to generate an unrooted phylogenetic tree as shown in **S1 Fig**.

### Ab2 rabbit antibody binding affinity and epitope characterization

To measure the binding affinity of Ab2 rabbit antibody, surface plasmon resonance (SPR) Biacore™-T200 instrument (GE Healthcare) was used. Series S sensor chip Protein A (29127555, GE Healthcare) was applied to capture each Ab2 rabbit IgG on a different flow cell (FC) to achieve approximately 100 response units (RU) followed by the injection of five-fold serial dilutions of Ab1 Fab (0.03 nM to 100 nM) in HBS-EP buffer (100 mM 4-(2-hydroxyethyl)-1-piperazineethanesulfonic acid (HEPES) pH 7.4, 150 mM NaCl, 3 mM EDTA, 0.05% (v/v) Surfactant P20) with a flow rate of 50 μl/min at 25°C. Association rates (k_on_) and dissociation rates (k_off_) were calculated using a simple one-to-one Langmuir binding model (Biacore T200 evaluation software version 2.0). The equilibrium dissociation constant (K_D_) was calculated as the ratio k_off_/k_on_.

To determine the epitope type of Ab2 rabbit antibody, the same captured format as described above was used. For Ag blocking or non-blocking epitope study, 100 nM Ab1 Fab was first injected for 5 min to reach saturation followed by the second injection of 50 nM Ag for 3 min to detect binding. The interactions between Ab1 Fab to Ab2 and the subsequent Ag to Ab1 Fab were recorded separately to calculate the differences in binding response (Ag-Ab1 Fab) as well as the theoretical Ag binding Rmax. Both factors were then used to determine the actual Ag binding R_max_ in percentage which is > 0 for Ag non-blocking epitope type and ≤ 0 for Ag blocking epitope type (**S1 Table**). For Ag and Ab1 complex specific epitope study, the binding R_max_ of 500 nM Ag + 50 nM Ab1 Fab complex and 50 nM Ab1 Fab only against similar level of protein A-captured Ab2 were recorded and the ratio between them was calculated in percentage. If the value was >10%, the Ab2 was considered as being of the Ag + Ab1 complex epitope type; otherwise, it belonged to non Ag + Ab1 complex epitope type (**S2 Table**).

For Ab2 rabbit antibody epitope binning study, the microarray-based 96 x 96 microfluidic system (IBIS-MX96 SPRi, Carterra USA) was used under a classical sandwich assay format. First, each Ab2 rabbit IgG (10 μg/mL in 10 mM sodium acetate buffer pH 4.5) was directly immobilized onto a sensor prism CMD 200M sensor chip (CMD 200M, XanTec Bioanalytics, Germany) using amine-coupling chemistry in the instrument of continuous flow microspotting (CFM, Carterra, USA). Next, 100 nM Ab1 Fab was injected over the sensor chip for 4 min binding followed by another 4 min injection of each Ab2 rabbit IgG (10 μg/mL in HBS-EP buffer) at 25°C. The chip surface was regenerated between each cycle using 10 mM Glycine pH 1.5 and the binding response was recorded and analyzed in Carterra microfluidics’ binning software for heat map generation and network plotting.

### Ab2 rabbit antibody for PK and ADA assay development

For PK assay development, a sandwich ELISA format was used. First, 96-well microtiter plate was separately coated with each Ab2 rabbit antibody (1 μg/mL) overnight at 4°C. Two-fold serial dilutions of therapeutic Ab1 antibody (20 ng/mL to 0.3 ng/mL) in assay buffer (1x PBS, 0.5% BSA, and 0.05% polysorbate 20) containing 2% pooled normal human serum were then added to the plate for 2 h incubation. To detect the bound therapeutic Ab1 antibody, each biotinylated version of Ab2 rabbit antibody (0.2 μg/mL; 10:1 biotinylation ratio) was added followed by streptavidin HRP conjugate (DY998, R&D Systems) and TMB substrate (5120–0047, KPL, Inc.) for color development. The plate reaction was stopped by adding 1 M phosphoric acid and absorbance was read at 450 nm with a 630 nm reference wavelength.

For ADA assay development, a bridging ELISA format was used. In this format, biotinylated and digoxigenin (DIG) conjugated therapeutic Ab1 reagents are bridged by ADAs. Positive control ADA samples were prepared by adding 1000 ng/mL of an individual Ab2 clone to neat human serum from healthy donors. The samples were diluted 1/20 followed by two-fold serial dilution to produce a titration curve (1000 ng/mL to 7.8 ng/mL). Biotinylated and DIG conjugated therapeutic Ab1 antibody reagents were added (each at 4 μg/mL; 10:1 challenge ratios) and incubated overnight with the diluted sample forming an immune complex with Ab2. Thereafter, the complex was captured onto a streptavidin-coated microtiter plate (11734776001, Roche) followed by detection using a mouse anti‑DIG HRP conjugated mAb (200032156, Jackson Immunoresearch) and color development as described above for the PK assay.

## Results

### Efficient workflow to isolate Ab1 specific single rabbit IgG^+^ B cell for *in vitro* clonal expansion culture

The extent of challenges associated with the isolation of antigen specific IgG^+^ B cells using different approaches has previously been reported [12]. The method of using fluorochrome-labeled antigens to sort out antigen-specific B cells with multi-parameter fluorescence activated cell sorting (FACS) was a promising approach. In order to improve the efficiency and to shorten the FACS sorting process time, we have developed a method to enrich IgG^+^ B cells before identifying antigen specific IgG^+^ B cells from peripheral blood mononuclear cells (PBMCs). Our approach efficiently eliminated non-IgG B cells including IgM B cells, myeloid cells, and T cells from PBMCs by Magnetic Cell Separation (MACS) beads-based negative-selection strategy. This negative-selection enrichment approach is believed to avoid potential activation-induced cell death and is more efficient than “dump channel” selection during FACS sorting to exclude non-IgG B cells. In our studies, it not only significantly increased the IgG^+^ B cell population up to 25-fold but also shortened the sorting time (3–30 mins versus 30–90 mins per plate) to potentially improve B cell survival rate. The following *in vitro* B cell culture process resulted in IgG concentrations range from 3–10 ug/mL, enabling subsequent binding and other types of functional assays. The resulting 10–100 B cells from single B cell culture process also enabled higher success rate for PCR and subsequent molecule cloning.

**Fig 1:**
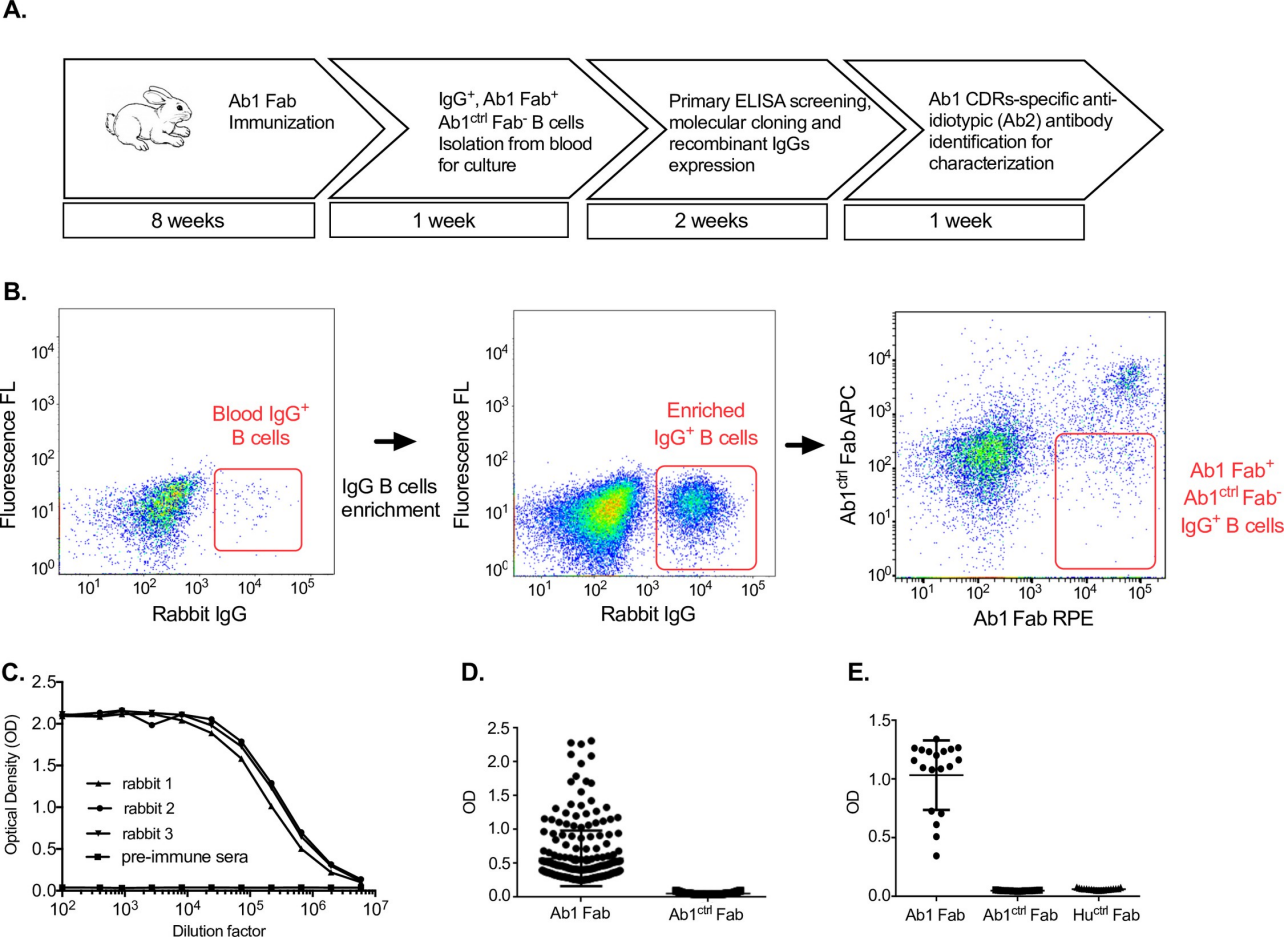
Ab1 CDRs-specific anti-IDs discovery from cultured rabbit B cells in project E. (**A**) Flowchart with timeline to isolate IgG^+^/Ab1 Fab^+^/ Ab1^ctrl^ Fab^-^ B cells from Ab1 Fab immunized rabbit peripheral blood for culture, primary ELISA screening, molecular cloning and recombinant IgGs expression to identify Ab1 CDRs-specific Ab2 for characterization (**B**) IgG^+^ B cells sorted out from immunized rabbit peripheral blood were further enriched for Ab1 Fab^+^/Ab1^ctrl^ Fab^-^/IgG^+^ B cells by Flow Cytometry (**C**) High serum titer of 3 rabbits against Ab1 Fab by ELISA (vs. pre-immunized serum control) demonstrated robust Ab1 immunization (**D**) High-throughput primary ELISA screening using cultured rabbit B cell supernatants confirmed majorities of clones were Ab1 Fab specific (OD > 0.25), but not Ab1^ctrl^ Fab (OD < 0.1) (**E**) Purified recombinant IgGs of 24 unique clones were reconfirmed specificity against Ab1 Fab but not to Ab1^ctrl^ Fab and other human control Fab (Hu^ctrl^ Fab) derived from native IgGs in normal human plasma. Ab1, antibody therapeutics; Ab2, anti-IDs.

Under project E, the workflow started with 8 weeks immunization of Ab1 Fab and 3 rabbits demonstrated strong anti-Ab1 serum IgG titer with positive binding at up to a 1:1,000,000 dilution factor (**Fig 1A, step 1;** and **Fig 1C**). By applying Ab1^ctrl^ Fab guided sorting strategy, B cells with Ab1 Fab^+^/Ab1^ctrl^ Fab^-^/IgG^+^ profiles were sorted individually into a 96-well plate filled with 200 ul supplemented RPMI 1640 culture medium and co-cultured with EL4-B5 feeder cells in conditional medium (rbTSN) for 1 week of *in vitro* clonal expansion (**Fig 1A, step 2;** and **Fig 1B**). The feeder cells provided CD40 ligand engagement and the rbTSN made from mitogen-PMA stimulated co-culture of rabbit thymocytes and monocytes supplied necessary cytokines for B cell proliferation and differentiation [11]. We observed that optimal B cell culture conditions are one of the key factors driving individual B cell survival rate and differentiation into antibody secreting plasma cells. The overall survival rate of IgG^+^ clones was 50–80% depending on the projects with an average IgG supernatant concentration of about 3–10 μg/mL. The supernatants derived from the cultured rabbit B cells are the most essential and valuable resources for the following step of primary enzyme-linked immunosorbent assay (ELISA) screening to confirm clones with desired Ab1 Fab^+^ and Ab1^ctrl^ Fab^-^ phenotype before molecular cloning (**Fig 1A, step 3**).

### High-throughput (HTP) screening to identify Ab1 specific clones for recombinant cloning

A robotic system that integrated multi-functional assays in one protocol was established in-house to screen Ab1 specific clones in an HTP manner. The system enabled us to run multiple antigen binding assays simultaneously to handle large panels of B cell culture supernatant (>50 96-well plates) screening in one day to identify positive hits. The advantages of this system is not only to provide a fast and robust antibody screening platform but also to eliminate unwanted clones and save downstream processing time.

In this project E, thousands of clone-cultured supernatants were subjected to primary ELISA screening and about 45% of clones were positive against Ab1 Fab (OD > 0.25) but not against Ab1^ctrl^ Fab (OD < 0.1) (**Fig 1D**). To save our cloning resources we decided to focus on the top 34 Ab2 clones which demonstrated significantly strong signal against Ab1 Fab binding (OD > 1) and a relative 10-fold lower signal against Ab1^ctrl^ Fab binding (OD < 0.1) for molecular cloning. The recombinant IgGs of 24 unique Ab2 clones were expressed after cloning; ELISA reconfirmed their Ab1 specificity by showing positive binding against Ab1 Fab (OD > 0.3), and nearly undetectable binding against designed Ab1^ctrl^ Fab (K1H3) and other native IgG Fab fragments derived from normal human plasma IgGs (Hu^ctrl^ Fab; 401116, Sigma) (OD < 0.05) (**Fig 1A, step 4** and **Fig 1E**).

### Designed Ab^ctrl^ Fab to guide Ab1 CDRs-specific B cell selection

**Fig 2:**
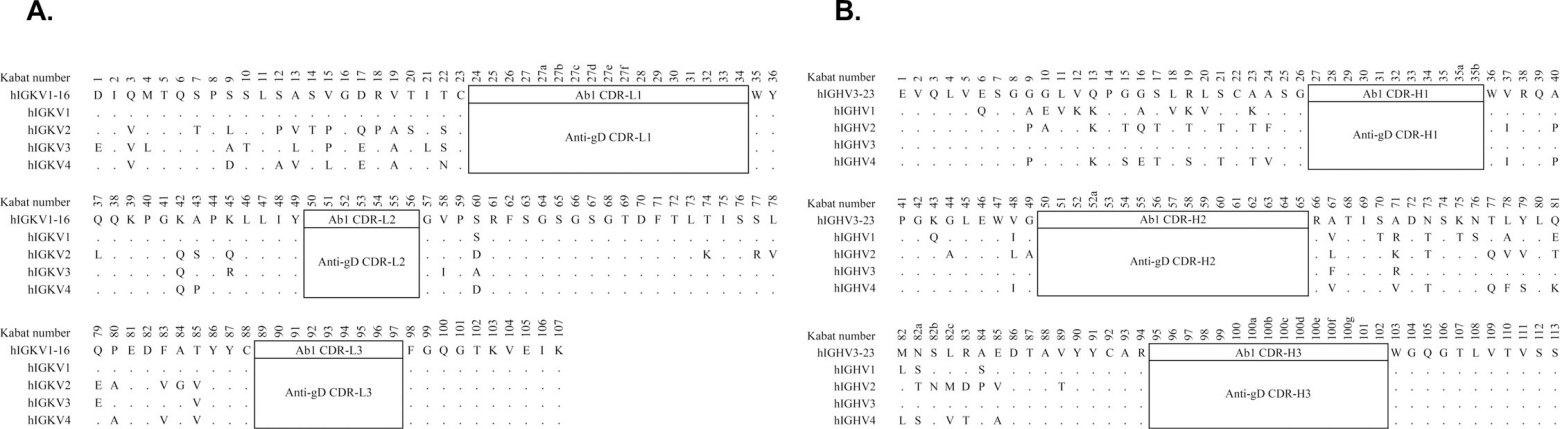
Designed Ab1 framework control used to guide Ab1 CDRs-specific anti-idiotypic antibodies (Anti-IDs) generation in project E. (**A**) The sequence of Ab1 light chain (LC) framework (hIGKV1-16) and Ab1 LC-CDRs was aligned with all 4 human IgG kappa LC frameworks and anti-gD LC-CDRs to indicate the different (letter) and identical (dot) amino acid residues. (**B**) The sequence of Ab1 heavy chain (HC) framework (hIGHV3-23) and Ab1 HC-CDRs was aligned with all 4 human IgG HC frameworks and anti-gD HC-CDRs to indicate the different (letter) and identical (dot) amino acid residues. The numbering at each position was by Kabat system and the CDRs were covered the regions of Kabat and Chothia definition in IMGT^15^. Ab1, antibody therapeutics.

The designed Ab1 framework control Fab (Ab1^ctrl^ Fab) was derived from individual sets of human framework germline genes by selecting the most prevalent amino acid residue at a given position. The CDRs of all Ab1^ctrl^ Fabs were mocked with an irrelevant anti-gD tag antibody to guide anti-IDs selection specifically towards Ab1 CDRs. In **Table 1**, the Ab1 light chain (LC) and heavy chain (HC) human framework germline from each project are shown in which the closest Ab1^ctrl^ Fab with the highest sequence identity was chosen to guide the selection. For example, both LC (hIGKV1-16) and HC (hIGHV3-23) of Ab1 framework germline were aligned with all 4 designed human LC Ab1^ctrl^ Fabs (hIGKV1-hIGKV4) and 4 human HC Ab1^ctrl^ Fabs (hIGHV1-hIGHV4), respectively, to compare the difference in sequences (**Fig 2A & 2B**). The hIGKV1(K1) and hIGHV3(H3) frameworks appeared to be the closest ones with 100% and 98% identity, respectively, as compared to Ab1 framework germline sequences. Consequently, we chose K1H3 as the appropriate Ab1^ctrl^ Fab for a negative gate to eliminate framework specific B cells in FACS sorting and a negative control in cultured supernatant primary ELISA screening (**Fig 1A, steps 2 & 3**).

### Anti-IDs affinity and epitope characterization

**Fig 3:**
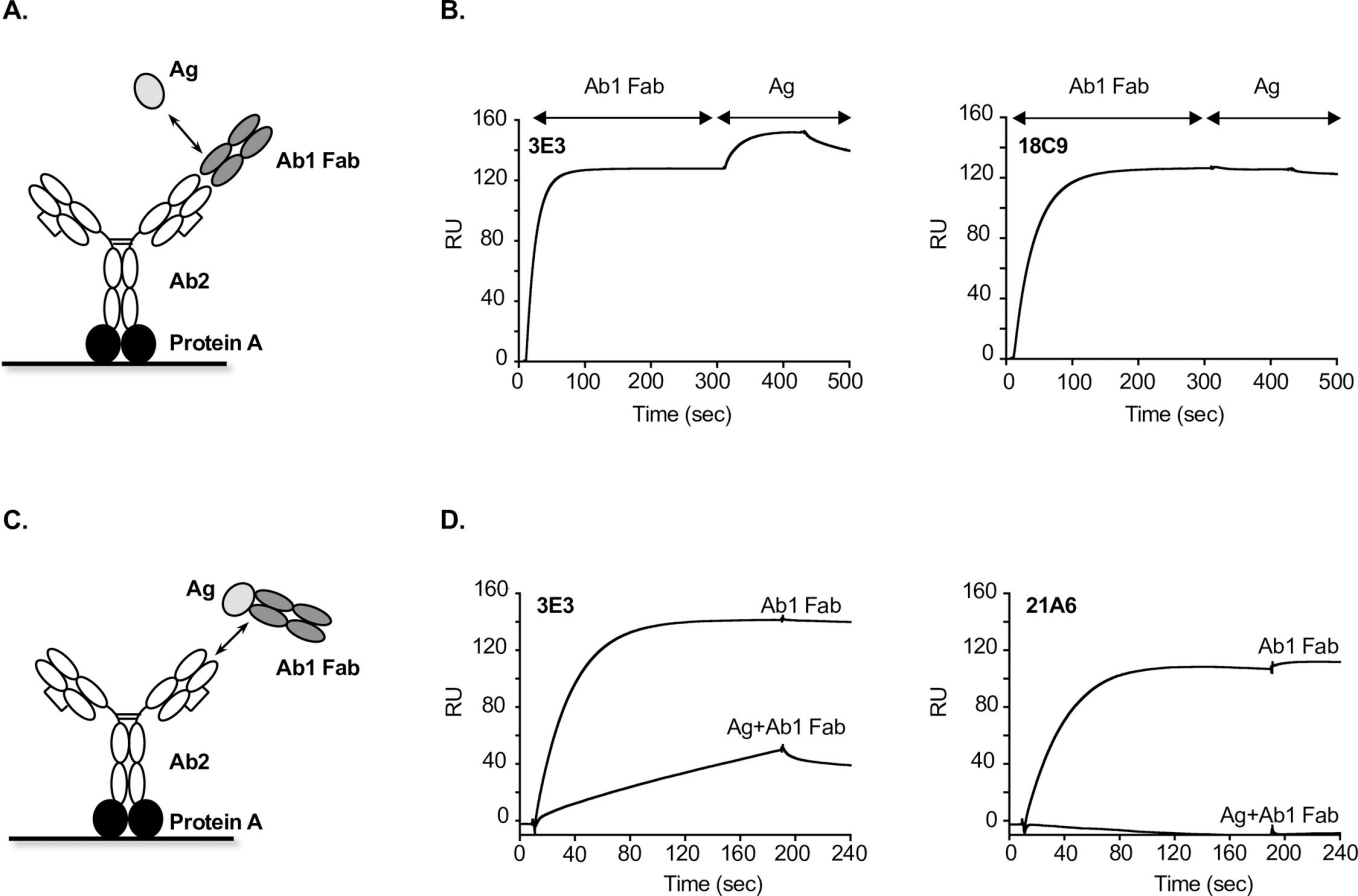
Anti-IDs epitope type characterization in project E. (**A**) Ag blocking or non-blocking epitope type determination format via Biacore SPR: Ab2 was first captured by protein A for Ab1 Fab binding followed by the detection of Ag binding (**B**) The Biacore sensorgrams illustrated that 3E3 is an Ag non-blocking anti-ID and 18C9 is an Ag blocking anti-ID (**C**) Ag and Ab1 complex epitope type determination format via Biacore SPR: Ab2 was first captured by protein A followed by the detection of Ag and Ab1 Fab complex binding (**D**) The Biacore sensorgrams illustrated that 3E3 is also capable of recognizing Ag and Ab1 complex whereas 21A6 is not. Ab1, antibody therapeutics; Ab2, anti-IDs; Ag, targets of antibody therapeutics.

High affinity anti-IDs are a desirable tool reagent in assay development for antibody therapeutics. In the bridging assay when low coating density of anti-IDs as capture reagents is required to avoid potential Ab1 binding to the surface with both arms (which would reduce assay sensitivity) the affinity of the anti-IDs is a determining factor to meet this requirement. In our studies, 6 out of 11 anti-IDs projects exceeded expectations by yielding multiple single-digit pM affinity antibodies with the remaining anti-IDs affinities in the mid to high pM affinity range (**Table 1**). In project E the majority of anti-IDs had affinities against the Fab-molecule of Ab1 < 0.5 nM (22 out of 24) with the best clone 14F9 having an affinity of 4 pM (**Table 2**). As Ab1 may interact with soluble or shed target molecules (Ag) in serum, there may be several forms of Ab1 present in circulation: either free, partially bound, or fully bound. Therefore, it is important to proactively establish what forms of Ab1 are to be measured in a given assay particularly when the molar concentration of Ag compared to Ab1 is not negligible at the timepoints of therapeutic concentration measurements. Using a highly-sensitivity HTP Biacore SPR, we classified various anti-ID epitope types according to their activity in the presence of Ag (**Fig 3)**. The Ag blocking type are anti-IDs that bind to the paratope of Ab1 and interfere with Ag binding thereby allowing for binding to Ab1 only when present with at least one free-Fab (e.g. 18C9 in **Fig 3B**). In contrast, an Ag non-blocking type anti-ID binds outside the paratope of Ab1 and still allows Ag to bind Ab1 and therefore can be used to detect free and possibly partially bound (one free Fab) and fully bound Ab1 (e.g. 3E3 in **Fig 3B**). To confirm if Ag non-blocking type of anti-IDs can also recognize the bound form of Ab1, we applied Ag + Ab1 complexes (premixed with 10-fold excess of Ag to saturate Ab1 binding sites) and compared its binding with that of Ab1 alone. Indeed, we identified a few anti-IDs (5 out of 19) that were capable of binding Ag + Ab1 complexes (e.g. 3E3 in **Fig 3D**) while the rest were not (e.g. 21A6 in **Fig 3D**).

**Table 2:** Anti-IDs binding affinity and epitope type characterization summary in project E.

Anti-IDs (Ab2)		Affinity to Ab1 [K_D_: nM]	Ab2 epitope type		
			Ag blocking	Ag non-blocking	Ag + Ab1 complex
**Group 1**	14F9	0.004	No	Yes	Yes
	14B11	0.028	No	Yes	Yes
	3E3	0.079	No	Yes	Yes
	21E2	0.083	No	Yes	Yes
	15A9	0.126	No	Yes	Yes
**Group 2**	2ID7	0.021	No	Yes	No
	1F9	0.023	No	Yes	No
	21A6	0.031	No	Yes	No
	23C7	0.055	No	Yes	No
	24B4	0.072	No	Yes	No
	9H10	0.073	No	Yes	No
	19C4	0.088	No	Yes	No
	23D4	0.098	No	Yes	No
	15A12	0.101	No	Yes	No
	28A4	0.108	No	Yes	No
	21F7	0.129	No	Yes	No
	20F6	0.131	No	Yes	No
	15C11	0.252	No	Yes	No
	27B5	0.366	No	Yes	No
**Group 3**	18C9	0.167	Yes	No	No
	28D6	0.432	Yes	No	No
	12A8	0.351	Yes	No	No
	19B2	5.688	Yes	No	No
	19F6	4.517	Yes	No	No

Twenty-four unique anti-IDs identified in project E are listed and ranked by their Biacore SPR affinity against Ab1 Fab (best to worst). The epitope type of each clone is included. In total there are 3 groups of anti-IDs; **Group 1:** Ag non-blocking with Ag + Ab1 complex specificity (5 clones), **Group 2:** Ag non-blocking without Ag + Ab1 complex specificity (14 clones), **Group 3:** Ag blocking (5 clones). Ab1, antibody therapeutics; Ab2, anti-IDs; Ag, targets of antibody therapeutics.

**Fig 4:**
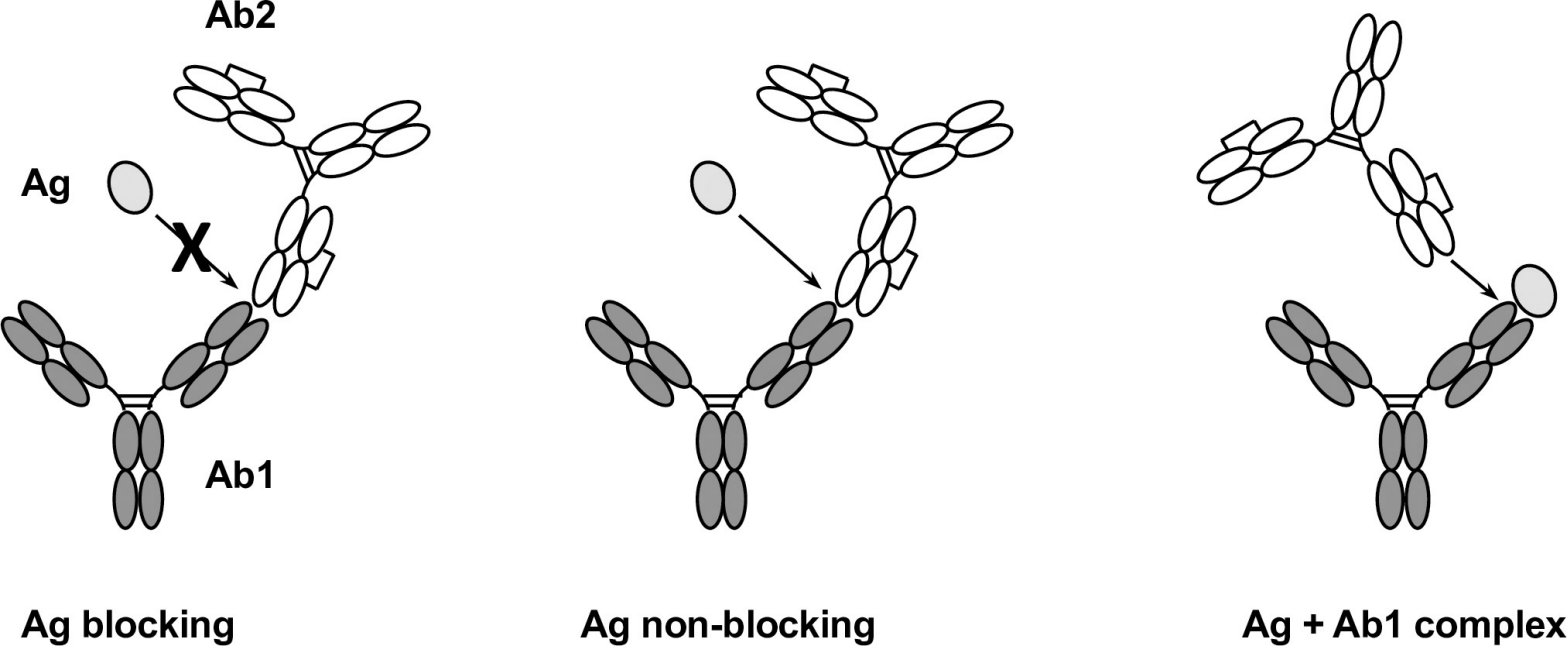
Three different types of anti-IDs in characterization. The Ag blocking anti-IDs (Ab2) bind the paratope of the drug (Ab1) inhibiting drug-target (Ag) binding for free drug detection. The Ag non-blocking anti-IDs bind outside the drug paratope allowing drug-target to bind the drug for free drug detection under drug-target interference. The Ag + Ab1 complex anti-IDs are specific for the drug-target complex exclusively for bound drug detection. Ab1, antibody therapeutics; Ab2, anti-IDs; Ag, targets of antibody therapeutics.

Overall, the 24 unique anti-IDs produced in project E can be categorized into three groups (**Fig 4** and **Table 2**): Group 1: Ag non-blocking with specificity to Ag + Ab1 complex (5 clones), Group 2: Ag non-blocking without specificity to Ag + Ab1 complex (14 clones), Group 3: Ag blocking (5 clones).

**Fig 5:**
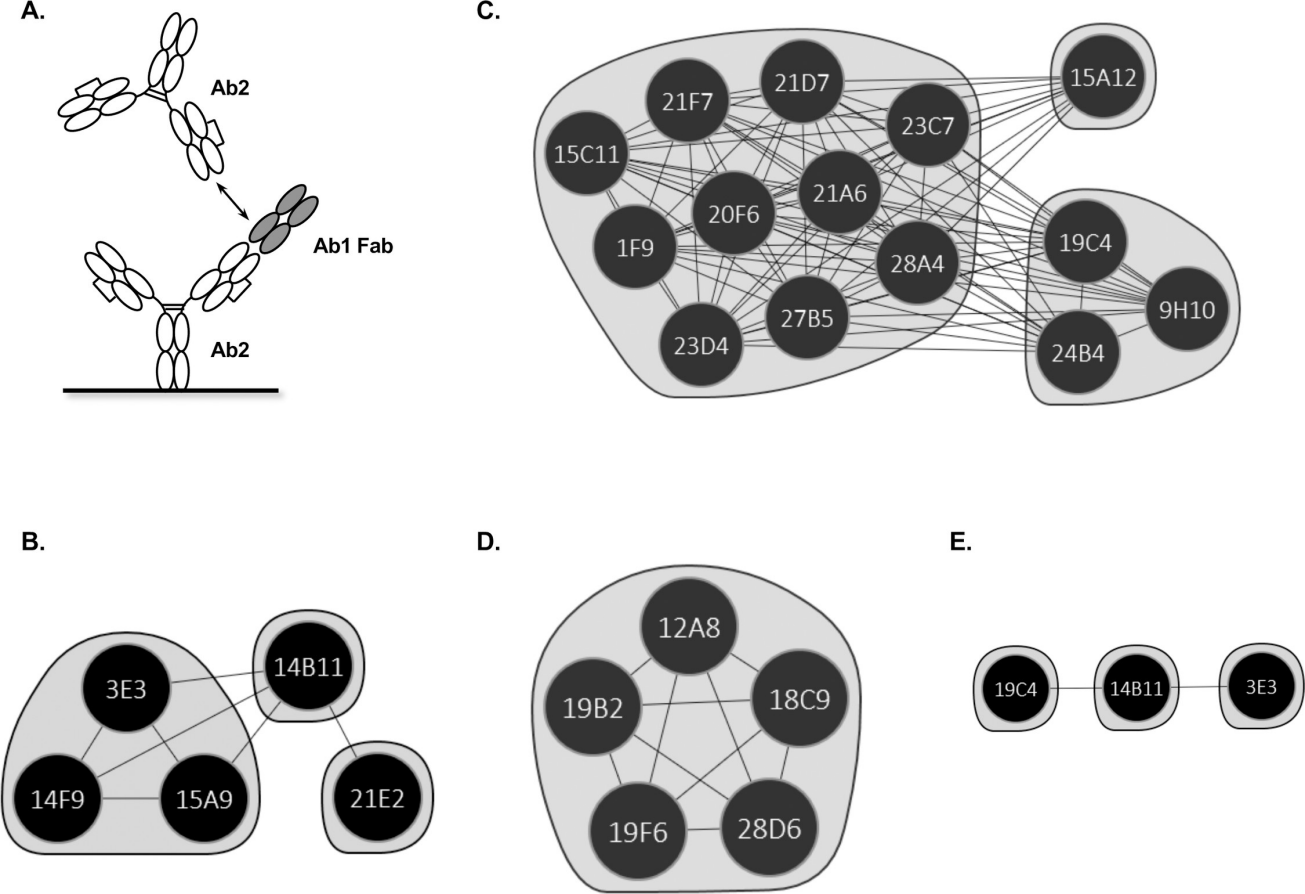
Anti-IDs epitope binning characterization in project E. (**A**) Anti-IDs epitope binning format in Carterra SPR microfluidics: The immobilized Ab2 was bound to Ab1 Fab first followed by the detection of pairwise Ab2 binding. The network plot depicting the epitope clusters deduced from binning a panel of 24 anti-IDs. The competing relationships (cords) between Ab2 (nodes) enable the anti-IDs to be clustered into bins (inscribed by the envelopes) where a bin represents a family of anti-IDs sharing an identical blocking profile when tested against the other anti-IDs. The plots are further categorized by epitope relationships as follows: (**B**) group 1 binders, (**C**) group 2 binders, (**D**) group 3 binders, and (**E**) both group 1 (3E3, 14B11) and group 2 (19C4) binders epitope relationships. Ab2, anti-IDs.

To elucidate the subtle epitope-binding differences in each group of anti-IDs, we further performed pairwise competition experiments with HTP Carterra SPR microfluidics using the classic sandwich format (**Fig 5A**). We observed the following binning format: in group 1 anti-IDs, three bins showed clone 14B11 binding to a bridging epitope between clone 21E2, 3E3, and others (**Fig 5B**). In group 2 anti-IDs, a similar result was observed except for a much larger group of anti-IDs (10 out of 14) targeting a bridging epitope (**Fig 5C**). In group 3 anti-IDs, interestingly, only one bin could be identified (**Fig 5D**).

### Anti-IDs for pharmacokinetics (PK) and anti-drug antibody (ADA) assay development

For PK and ADA assay development in project E, we selected 5 anti-IDs, two from group 1 (3E3 and 14B11) and three from group 2 (9H10, 19C4, and 24B4), aiming to measure total Ab1. An important consideration in the context of assay development decisions for this project was the fact that the Ag was expected to be present in study samples at non-negligible concentrations compared to Ab1 at the time of PK measurements.

**Fig 6:**
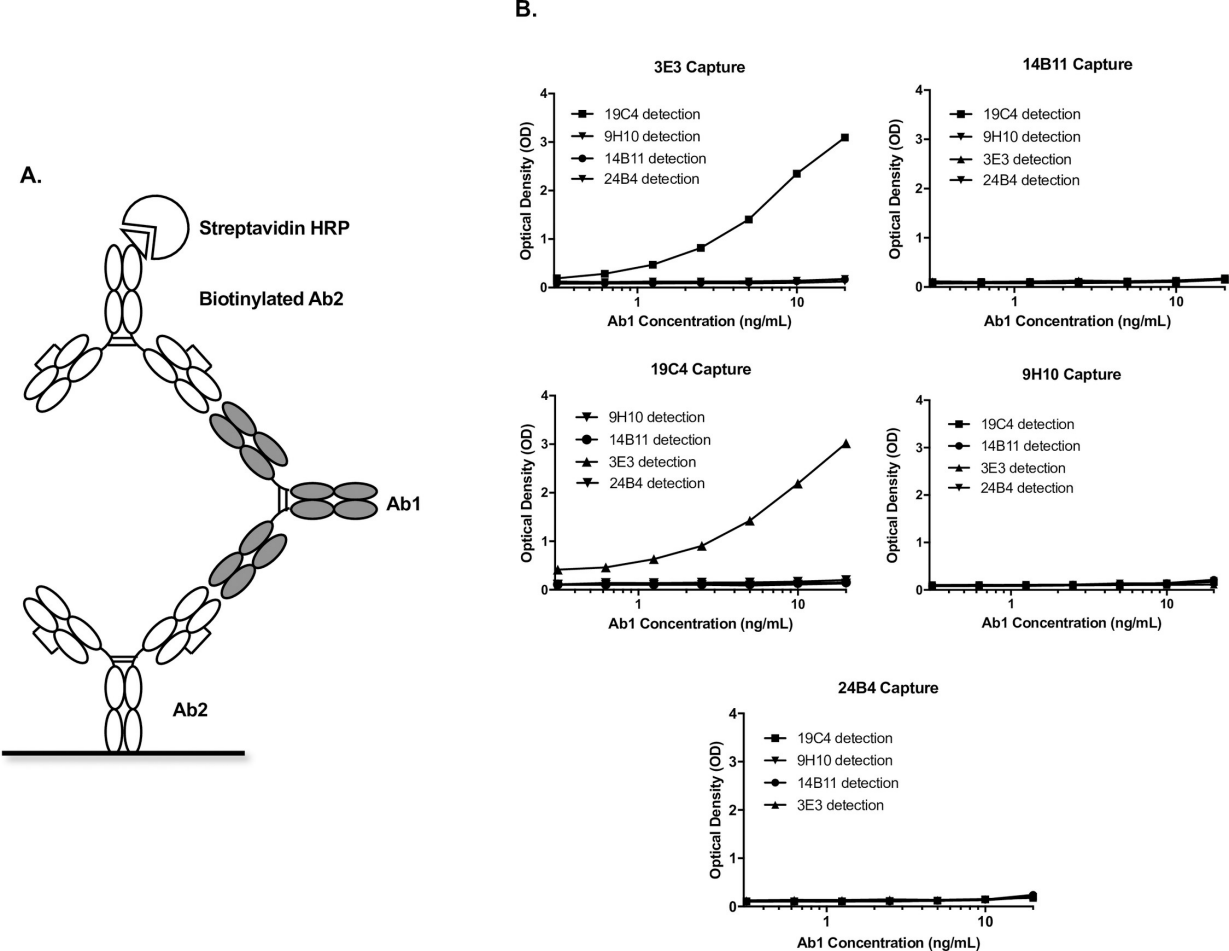
Anti-IDs pharmacokinetic (PK) assay development in project E. (**A**) Sandwich ELISA format for PK assay development: Each Ab2 antibody was immobilized on the ELISA plate to serve as Ab1 capture reagent while the other Ab2 antibodies were biotinylated to serve as the detection reagent with streptavidin HRP conjugate (**B**) Clones 3E3 (group 1) and 19C4 (group 2) are a suitable capture and detection antibody pair to produce robust dose-response curves with high signal-to-noise ratios for PK assay development. In contrast, clone 14B11 (group 1), 9H10 & 24B4 (group 2) do not work in any combination with others as capture or detection reagents due to the lack of indicated signal. Ab1, antibody therapeutics; Ab2, anti-IDs.

Using the sandwich ELISA format shown in **Fig 6A**, 3E3 (group 1) and 19C4 (group 2) formed a suitable antibody pair to capture and detect Ab1 (**Fig 6B**). The pair displayed a high signal-to-noise ratio and robust dose-response curve titration in either orientation. As evidenced by epitope binning characterization studies, 3E3 and 19C4 have distinct and non-overlapping epitopes allowing for both reagents to sandwich Ab1 (**Fig 5E**). In contrast, clones 14B11 (group 1), 9H10 and 24B4 (group 2) did not demonstrate compatibility with either 3E3 or 19C4. Clones 9H10 and 24B4 have non-overlapping epitopes with clone 3E3; however, neither offer the same robust dose-response curve when paired with 3E3 as 19C4 does despite sharing similar characteristics with 19C4. These results underscore the importance of experimental bioanalytical data in this type of reagent selection process complementing the reagent characterization data. While coating the plate with either 3E3 or 19C4 and using the other clone as detection reagent results in an acceptable assay, we chose to use 3E3 as the capture antibody due to its lower background and better signal-to-noise ratio. This format also favors capture of Ab1-Ag complexes, increasing the assay’s ability to measure bound-drug.

**Fig 7:**
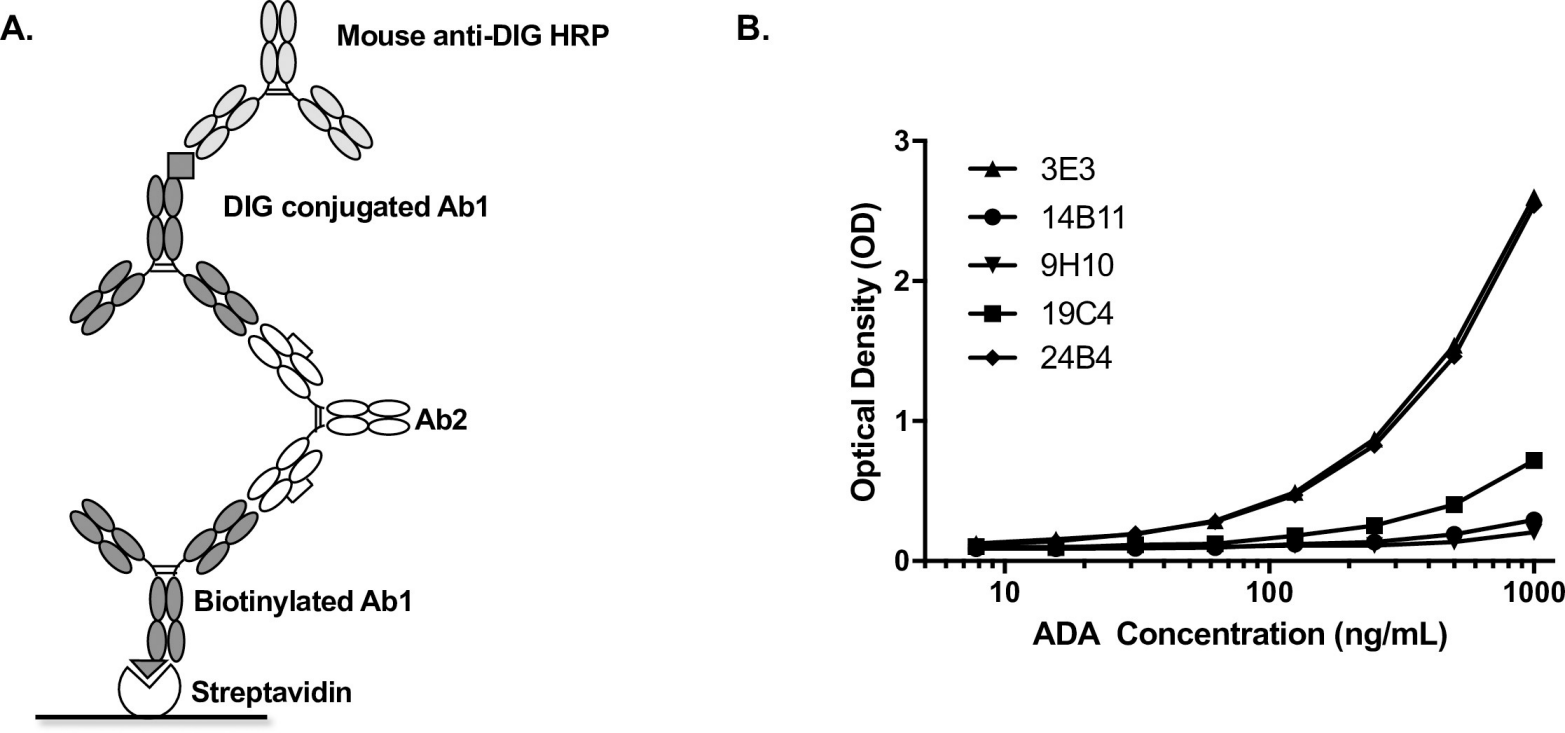
Anti-IDs anti-drug antibody (ADA) assay development in project E. (**A**) Bridging ELISA format for ADA assay development: each Ab2 antibody was tested on the ELISA microtiter plate to bridge streptavidin-captured biotinylated Ab1 and DIG conjugated Ab1 with mouse anti-DIG horseradish peroxidase (HRP) conjugate as detection reagent (**B**) Titration of individual Ab2 clone indicated that both clones 3E3 and 24B4 provided the greatest signal to noise ratio for suitable surrogate positive ADA control reagents. Ab1, antibody therapeutics.

A sensitive assay to detect the presence of ADA in treated patients is critically important for evaluating immune responses to the recombinant therapeutics. For ADA assay development, anti-IDs should preferably target epitopes that are unique to drug molecules. Antibodies against the therapeutics derived from B-cells using the production and selection strategy described here are most likely anti-IDs. Therefore, we explored whether any of the aforementioned five clones could also serve as a surrogate positive control in the clinical immunogenicity assay for project E. Using the bridging ELISA format shown in **Fig 7A**, we observed that both 3E3 and 24B4 produced a specific and robust binding curve, with superior characteristics over the other anti-IDs indicating that they would be suitable anti-ID reagents for ADA assay development in this project (**Fig 7B**).

## Discussion

Anti-IDs are invaluable reagents for PK and ADA assays required for bioanalytical support of therapeutic drugs in preclinical development as well as during clinical trials. High sensitivity and specificity, strong affinity, as well as well-defined epitope-binding specificity are important features for this type of reagent antibody. The rabbit single B cell sorting-culture and cloning technology described herein enabled us to consistently, efficiently, and productively generate panels of monoclonal anti-IDs with desired properties in a variety of mAb therapeutic projects while meeting stringent bioanalytical assay performance requirements. By incorporating a negative-selection step with the Ab1^ctrl^ Fab designed to mimic the therapeutic drugs’ framework, we were able to considerably enhance our ability to selectively produce highly specific anti-IDs against the unique amino acid sequences of therapeutic-drug CDRs. Anti-IDs specifically directed to the CDRs of mAb therapeutic drugs (often humanized or human antibodies) are less prone to interference from excess amounts of endogenous human IgGs (with similar Ig framework as the drug) present in biological matrices such as human serum. The concept of deploying an Ab1^ctrl^ Fab design combining 4 human IgG kappa LC families (Kappa 1-Kappa 4) and 4 human HC families (VH1-VH4) was shown here to be highly beneficial and readily applicable to other antibody families such as lambda LC and other HC for anti-IDs discovery framework control.

In this study, we described practical solutions aimed to address common difficulties often encountered in anti-ID antibody discovery including the low immunogenicity of therapeutic drug CDRs, unwanted reactivity against the constant regions of the antibody as well as weak affinity and low-yield batches of potential anti-ID candidates. To overcome these obstacles, we chose the Fab-immunogen approach to not only avoid presentation of constant HC CH2 and CH3 as antigenic determinants during immunization but also prevent Fc sequences from triggering non-specific interactions during rabbit B cell sorting. In some projects (B/D/E/F/G/I), we noticed lower anti-ID yields after primary ELISA screening possibly due to low immunogenicity of the CDRs for those targets. However, with the power of rabbit single B cell sorting-culture and cloning technology, we were able to compensate for the weak immune response in these projects by delivering sufficient number of anti-IDs with diversified functions adequately supplying downstream assay development efforts as exemplified in project E case-study. The strong monovalent binding affinities of the majority of anti-IDs generated using this platform in all 11 projects ranging from pM to low single digit nM were sufficiently sensitive to meet assay performance requirements without further affinity maturation.

Having a panel of well-characterized anti-IDs can be helpful in the development of both clinical PK and ADA assays in many ways. For PK assays availability of multiple anti-ID clones allows for assessment and comparison between various assay formats including the anti-ID/anti-ID bridging format described herein. This format, which is preferred for the development of free-drug PK assays when using Group 2-type anti-IDs, can improve both sensitivity and specificity of the assay over the use of generic non-drug specific reagents [19]. Similarly, Group 1-type anti-IDs can be leveraged for total-drug PK assays, due to their ability to bind pre-formed drug-Ag complexes. Furthermore, the use of specific reagents contributes to a robust dose-response curve covering a wide dynamic range of the assay while maintaining acceptable accuracy and precision [20]. However, clones sharing similar characteristics may perform differently in the assay (as demonstrated with clones 9H10 and 24B4). This can be due to varying degrees of plate-coating efficiencies between anti-IDs, structural changes introduced during the conjugate-formation process or matrix effects (e.g. interfering factors present in blood, plasma, serum, etc.). Therefore, an adequately diverse panel of clones available for screening and selection may be a critical element for successful anti-ID reagent production programs. Epitope characterization and grouping can further inform assay development decisions and provide a choice between development of a total-drug assay or a free-drug assay as best suited for specific project needs.

Selection criteria of anti-IDs for ADA assays on the other hand are often different from those applied for PK assays. Beyond controlling an assay’s performance over time as a surrogate positive ADA source, anti-ID is used to demonstrate and assess important assay parameters during validation such as sensitivity, specificity, drug tolerance, precision, and analyte stability. Furthermore, anti-IDs may also be used to characterize antibody responses against particular epitopes for their therapeutic use in competition assays.

In conclusion, we have successfully established a novel, robust, and reliable in-house anti-ID discovery strategy and platform using rabbit single B cell sorting-culture and cloning technology. In just 12 weeks, we were able to generate a large and diverse panel of functionally active monoclonal anti-IDs against the CDRs of a variety of therapeutic drug candidates thus providing critical support to multiple bioanalytical projects in our development pipeline. These anti-ID panels provide great versatility for specialized assay development in research, preclinical, and clinical development. Unlike polyclonal serum antibodies which tend to have lot-to-lot variability and supply limitations, all cloned monoclonal anti-ID antibodies can be recombinantly expressed at the desired time and at an appropriate scale. The optimized rabbit single B cell sorting-culture and cloning technology has broad applicability for antibody discovery. Here, we have successfully demonstrated that a similar approach can also be used to generate high quality rabbit mAbs directed at therapeutic drugs providing a valuable source of uniquely useful critical reagents for bioanalytical support of mAb therapeutic development programs.

## Supporting information

S1 Fig Phylogenetic tree of the 24 unique clone VH:VL CDRs concatenated strings in project E. The unrooted phylogenetic tree visualizes the CDR differences between the 24 clones. The scale bar represents 4% sequence difference and the individual sequence differences are accordingly labelled on each branch. The clones are also color-coded according to the grouping designation described in **Table 2** where red is group 1, green is group 2, and blue is group 3. It can be observed that two clades towards the bottom of the tree consist predominantly group 2 clones implicating that these could be affinity matured variants originating from common ancestral clones. On the other hand, both group 1 and 3 clones tend to be derived independently resulting in scattered locations along the tree.
(TIF)

S1 TableAnti-IDs Ag blocking or non-blocking epitope type determination in project E.The binding response of Ab1 Fab against each protein A-captured Ab2 clone following Ag binding to Ab1 Fab was recorded. The actual Ag binding response (Ag—Ab1 Fab) was used to determine the actual Ag binding Rmax [%] by dividing it with the theoretical Ag binding Rmax; Rmax = $\frac{molecular\;weight\;of\;Ag}{molecular\;weight\;of\;Ab1\;Fab}X\;(Ab1\;Fab\;binding\;response)\;X\;(Ab1\;Fab\;stoichiometry)$; The Ag blocking or non-blocking epitope type of Ab2 is defined by the actual Ag binding Rmax_x_ [%], which is >0 for Ag non-blocking and ≤0 for Ag blocking. All 24 unique anti-IDs identified in project E are listed and ranked by their actual Ag binding Rmax, [%] (highest to lowest).(DOCX)

S2 TableAnti-IDs Ag and Ab1 complex epitope type determination in project E.Under similar protein A capturing level for each Ab2 clone the binding Rmax of Ag + Ab1 Fab as well as Ab1 Fab were recorded. The binding Rmax ratio between Ag + Ab1 Fab and Ab1 Fab was calculated as %. If the value was >10%, the clone was considered Ag and Ab1 complex specific epitope type. All 24 unique anti-IDs identified in project E are listed and ranked by their binding Rmax ratio ($\frac{\left(Ag+Ab1\;Fab\right)}{Ab1\;Fab}$) [%] (highest to lowest).(DOCX)
