# Supplementary figures and images for: Rapid identification of anti-idiotypic mAbs with high affinity and diverse epitopes by rabbit single B-cell sorting-culture and cloning technology
Source: PLoS One. 2020 Dec 21;15(12):e0244158. doi: 10.1371/journal.pone.0244158 (PMC7751967; doi:10.1371/journal.pone.0244158)

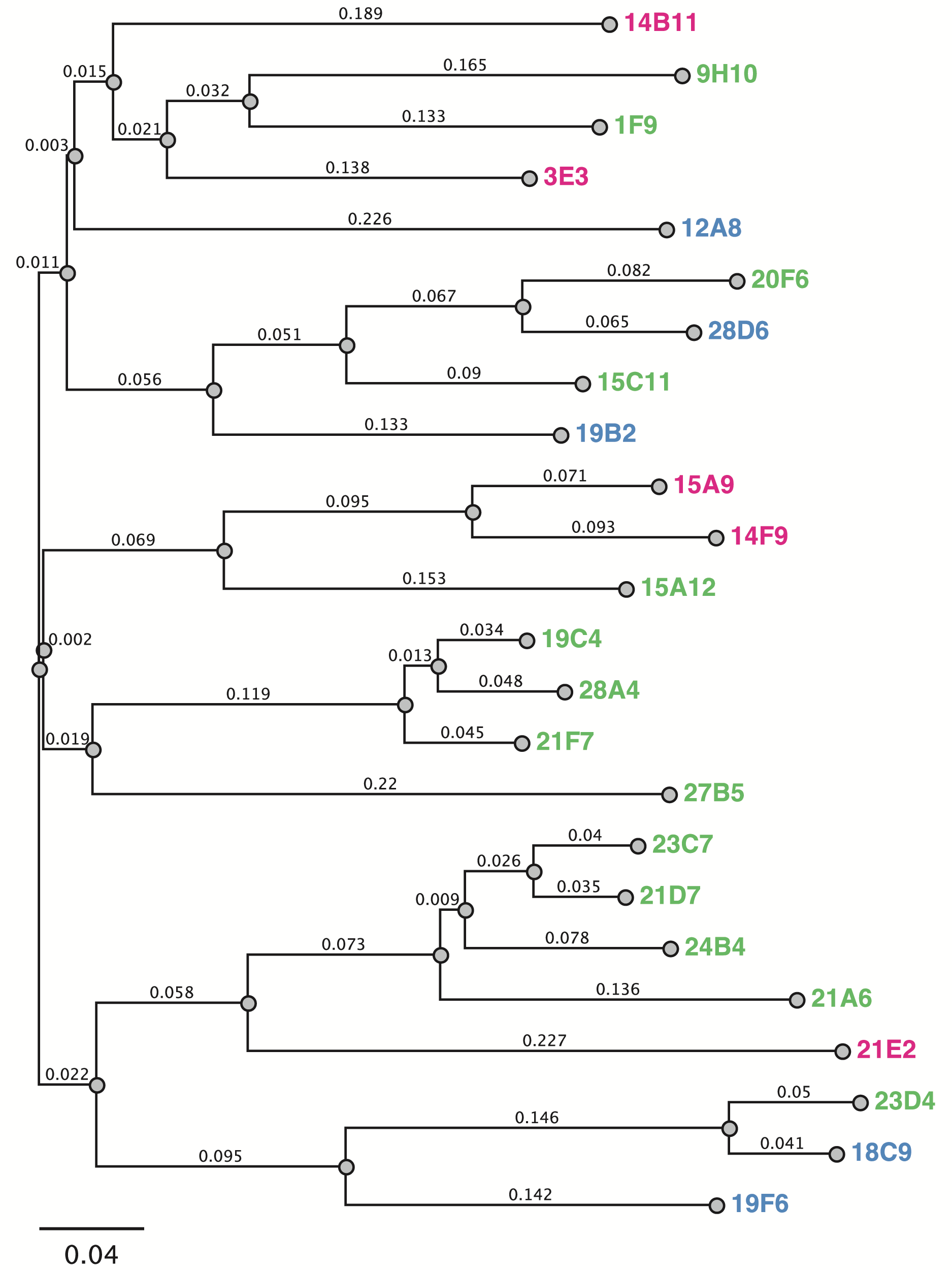

Supplement: S1 Fig — The unrooted phylogenetic tree visualizes the CDR differences between the 24 clones. The scale bar represents 4% sequence difference and the individual sequence differences are accordingly labelled on each branch. The clones are also color-coded according to the grouping designation described in Table 2 where red is group 1, green is group 2, and blue is group 3. It can be observed that two clades towards the bottom of the tree consist predominantly group 2 clones implicating that these could be affinity matured variants originating from common ancestral clones. On the other hand, both group 1 and 3 clones tend to be derived independently resulting in scattered locations along the tree. (TIF) [file pone.0244158.s001.tif]
