# Supplementary material for: Rapid identification of anti-idiotypic mAbs with high affinity and diverse epitopes by rabbit single B-cell sorting-culture and cloning technology
Source: PLoS One. 2020 Dec 21;15(12):e0244158. doi: 10.1371/journal.pone.0244158 (PMC7751967; doi:10.1371/journal.pone.0244158)
Supplement: S1 Table — The binding response of Ab1 Fab against each protein A-captured Ab2 clone following Ag binding to Ab1 Fab was recorded. The actual Ag binding response (Ag—Ab1 Fab) was used to determine the actual Ag binding Rmax [%] by dividing it with the theoretical Ag binding Rmax; Rmax = molecularweightofAgmolecularweightofAb1FabX(Ab1Fabbindingresponse)X(Ab1Fabstoichiometry); The Ag blocking or non-blocking epitope type of Ab2 is defined by the actual Ag binding Rmaxx [%], which is >0 for Ag non-blocking and ≤0 for Ag blocking. All 24 unique anti-IDs identified in project E are listed and ranked by their actual Ag binding Rmax, [%] (highest to lowest). (DOCX) [file pone.0244158.s002.docx]

**S1 Table. Anti-IDs Ag blocking or non-blocking epitope type determination in project E.**

| **Anti-IDs (Ab2)** | **Affinity to Ab1 Fab [K_D_: nM]** | **Ab1 Fab binding response [RU]** | | **Ag binding response [RU]** | **Ag –Ab1 binding response [RU]** | | **Theoretical Ag binding Rmax [RU]** | | **Actual Ag binding  Rmax [%]** | **Ab2 epitope type**  **(Ag blocking)** |
| --- | --- | --- | --- | --- | --- | --- | --- | --- | --- | --- |
| 3E3 | 0.079 | 128 | 152 | | | 24 | 64 | 38 | | No |
| 21E2 | 0.083 | 115 | 126 | | | 11 | 58 | 19 | | No |
| 15A12 | 0.101 | 125 | 136 | | | 11 | 63 | 18 | | No |
| 14B11 | 0.028 | 138 | 150 | | | 12 | 69 | 17 | | No |
| 14F9 | 0.004 | 128 | 137 | | | 9 | 64 | 14 | | No |
| 9H10 | 0.073 | 95 | 101 | | | 6 | 48 | 13 | | No |
| 19C4 | 0.088 | 124 | 132 | | | 8 | 62 | 13 | | No |
| 23D4 | 0.098 | 128 | 136 | | | 8 | 64 | 13 | | No |
| 21F7 | 0.129 | 120 | 128 | | | 8 | 60 | 13 | | No |
| 20F6 | 0.131 | 124 | 132 | | | 8 | 62 | 13 | | No |
| 27B5 | 0.366 | 132 | 140 | | | 8 | 66 | 12 | | No |
| 1F9 | 0.023 | 122 | 129 | | | 7 | 61 | 11 | | No |
| 21A6 | 0.031 | 140 | 148 | | | 8 | 70 | 11 | | No |
| 24B4 | 0.072 | 131 | 138 | | | 7 | 66 | 11 | | No |
| 28A4 | 0.108 | 128 | 135 | | | 7 | 64 | 11 | | No |
| 15C11 | 0.252 | 110 | 116 | | | 6 | 55 | 11 | | No |
| 23C7 | 0.055 | 149 | 154 | | | 5 | 75 | 7 | | No |
| 21D7 | 0.021 | 184 | 189 | | | 5 | 92 | 5 | | No |
| 15A9 | 0.126 | 155 | 157 | | | 2 | 78 | 3 | | No |
| 18C9 | 0.167 | 126 | 126 | | | 0 | 63 | 0 | | Yes |
| 28D6 | 0.432 | 110 | 110 | | | 0 | 55 | 0 | | Yes |
| 12A8 | 0.351 | 146 | 144 | | | -2 | 73 | -3 | | Yes |
| 19B2 | 5.688 | 117 | 111 | | | -6 | 59 | -10 | | Yes |
| 19F6 | 4.517 | 121 | 101 | | | -20 | 61 | -33 | | Yes |

The binding response of Ab1 Fab against each protein A-captured Ab2 clone following Ag binding to Ab1 Fab was recorded. The actual Ag binding response (Ag - Ab1 Fab) was used to determine the actual Ag binding Rmax [%] by dividing it with the theoretical Ag binding Rmax; Rmax = $\frac{molecular weight of Ag}{molecular weight of Ab1 Fab} X \left( Ab1 Fab binding response \right) X \left( Ab1 Fab stoichiometry \right)$; The Ag blocking or non-blocking epitope type of Ab2 is defined by the actual Ag binding Rmax [%], which is >0 for Ag non-blocking and ≤0 for Ag blocking. All 24 unique anti-IDs identified in project E are listed and ranked by their actual Ag binding Rmax [%] (highest to lowest).
