# Supplementary material for: Rapid identification of anti-idiotypic mAbs with high affinity and diverse epitopes by rabbit single B-cell sorting-culture and cloning technology
Source: PLoS One. 2020 Dec 21;15(12):e0244158. doi: 10.1371/journal.pone.0244158 (PMC7751967; doi:10.1371/journal.pone.0244158)
Supplement: S2 Table — Under similar protein A capturing level for each Ab2 clone the binding Rmax of Ag + Ab1 Fab as well as Ab1 Fab were recorded. The binding Rmax ratio between Ag + Ab1 Fab and Ab1 Fab was calculated as %. If the value was >10%, the clone was considered Ag and Ab1 complex specific epitope type. All 24 unique anti-IDs identified in project E are listed and ranked by their binding Rmax ratio ((Ag+Ab1Fab)Ab1Fab) [%] (highest to lowest). (DOCX) [file pone.0244158.s003.docx]

**S2 Table. Anti-IDs Ag and Ab1 complex epitope type determination in project E.**

| **Anti-IDs (Ab2)** | **Ag + Ab1 Fab binding Rmax [RU]** | **Ab1 Fab binding Rmax [RU]** | **(Ag + Ab1 Fab)/Ab1 Fab binding Rmax [%]** | **Ab2 epitope type (Ag + Ab1 complex)** |
| --- | --- | --- | --- | --- |
| 3E3 | 47 | 143 | 33 | Yes |
| 14B11 | 25 | 147 | 17 | Yes |
| 21E2 | 26 | 194 | 13 | Yes |
| 14F9 | 17 | 145 | 12 | Yes |
| 15A9 | 17 | 164 | 10 | Yes |
| 20F6 | 7 | 124 | 6 | No |
| 15A12 | 6 | 123 | 5 | No |
| 23D4 | 6 | 138 | 4 | No |
| 27B5 | 6 | 139 | 4 | No |
| 21D7 | 6 | 162 | 4 | No |
| 19B2 | 3 | 106 | 3 | No |
| 1F9 | 4 | 142 | 3 | No |
| 12A8 | 2 | 164 | 1 | No |
| 23C7 | 2 | 172 | 1 | No |
| 21A6 | 0 | 155 | 0 | No |
| 24B4 | -1 | 151 | -1 | No |
| 15C11 | -5 | 120 | -4 | No |
| 19C4 | -7 | 136 | -5 | No |
| 28D6 | -7 | 129 | -5 | No |
| 18C9 | -6 | 115 | -5 | No |
| 28A4 | -10 | 157 | -6 | No |
| 21F7 | -9 | 138 | -7 | No |
| 9H10 | -7 | 94 | -7 | No |
| 19F6 | -9 | 119 | -8 | No |

Under similar protein A capturing level for each Ab2 clone, the binding Rmax of Ag + Ab1 Fab as well as Ab1 Fab were recorded. The binding Rmax ratio between Ag + Ab1 Fab and Ab1 Fab was calculated as %. If the value was >10%, the clone was considered Ag and Ab1 complex specific epitope type. All 24 unique anti-IDs identified in project E are listed and ranked by their binding Rmax ratio ( $\frac{(Ag +Ab1 Fab)}{Ab1 Fab}$) [%] (highest to lowest).
